# Accidents involving motorcycles and potential years of life lost. An ecological and exploratory study

**DOI:** 10.1590/1516-3180.2017.0098070817

**Published:** 2018-01-15

**Authors:** Drielle Rezende Pavanitto, Renata Armani de Moura Menezes, Luiz Fernando Costa Nascimento

**Affiliations:** I Medical Student, Department of Medicine, Universidade de Taubaté (UNITAU), Taubaté (SP), Brazil.; II PhD. Researcher, Department of Energy, Universidade Estadual Paulista (UNESP), Guaratinguetá (SP), and Assistant Professor, Department of Medicine, Universidade de Taubaté (UNITAU), Taubaté (SP), Brazil.

**Keywords:** Life expectancy, Geographic information systems, Motorcycles, Accidents, traffic

## Abstract

**CONTEXT AND OBJECTIVE:**

Traffic accidents have gained prominence as one of the modern epidemics that plague the world. The objective of this study was to identify the spatial distribution of potential years of life lost (PYLL) due to accidents involving motorcycles in the state of São Paulo, Brazil.

**DESIGN AND SETTING:**

Ecological and exploratory study conducted in São Paulo.

**METHODS:**

Data on deaths among individuals aged 20-39 years due to motorcycle accidents (V20-V29 in the International Classification of Diseases, 10^th^ revision) in the state of São Paulo in the years 2007-2011 were obtained from DATASUS. These data were stratified into a database for the 63 microregions of this state, according to where the motorcyclist lived. PYLL rates per 100,000 inhabitants were calculated. Spatial autocorrelations were estimated using the Global Moran index (IM). Thematic, Moran and Kernel maps were constructed using PYLL rates for the age groups of 20-29 and 30-39 years. The Terraview 4.2.2 software was used for the analysis.

**RESULTS:**

The PYLL rates were 486.9 for the ages of 20-29 years and 199.5 for 30-39 years. Seventeen microregions with high PYLL rates for the age group of 20-29 years were identified. There was higher density of these rates on the Kernel map of the southeastern region (covering the metropolitan region of São Paulo). There were no spatial autocorrelations between rates.

**CONCLUSIONS:**

The data presented in this study identified microregions with high accident rates involving motorcycles and microregions that deserve special attention from regional managers and traffic experts.

## INTRODUCTION

Brazil occupies the fifth place among the countries with the highest numbers of traffic deaths, only preceded by India, China, the United States and Russia. The mortality rate due to road transport accidents (RTAs) increased from 18.0 to 22.5 deaths/100,000 inhabitants between 2000 and 2010, which represented an increase of 32.3% over this decade.^1^ The motorcycle accident mortality rate in Brazil increased from 0.5 to 4.5/100,000 inhabitants from 1996 to 2009, which represented an increase of 800% over this period.^2^ Collisions between motorcycles and automobiles was the largest cause of these accidents (38.4%) and accidental falls from motorcycles was the second (32%), in a survey on 378 recorded incidents involving motorcycle accidents in Corumbá, Mato Grosso do Sul, in 2010.^3^ Motorcycle accidents are highly worrisome, since they mainly affect young people of economically active age, with a high chance of death,^4^ as shown in the state of Sergipe, among 554 motorcycle accident victims who were attended at a referral center.^5^

In the western region of the Paraíba valley, in the state of São Paulo, a cluster of nine municipalities with the highest hospitalization rates due to motorcycle accidents was identified through spatial analysis.^6^ This approach, including the indicator of potential years of life lost (PYLL), was used in the state of Pernambuco, in the year 2007, for a study on accidents due to RTAs. It was found that 13,196 RTAs involved motorcycles, which represented 27. 5% of the total number of accidents.^7^ The PYLL indicator estimates the impact of deaths on society. It qualifies deaths and presents a new criterion for selecting priorities. Through a single figure, the PYLL indicator covers the magnitude of the impact, expressed as the number of deaths according to the age at which the event occurs, and the vulnerability to death. Thus, this indicator quantifies mortality not only according to the frequency of occurrence of death but also according to the time left to live, up to the limit of life expectancy.

Spatial analysis is a geostatistical tool that is becoming widely used in research, including in health-related fields. One of its applications encompasses identification of spatial clusters, i.e. any aggregations of events that are not merely random. Through developing maps, the aim is to analyze whether the distribution of these events actually presents a spatial pattern, or whether it is random. In addition, these data can be used by other sectors, such as transport-related education and inspection, and may serve as a basis for reducing the risk of accidents. Thus, one of the indications for using spatial analysis is to identify sites with greater intensity of accidents, i.e. critical areas. This can serve as backing for implementation of prevention and control measures, as presented in several recent articles published in Brazil.^6^^,^^8^^,^^9^^,^^10^^,^^11^

## OBJECTIVE

Considering the increasing number of motorcycles that form part of the vehicle fleet, the vulnerability of motorcyclists and their possible pillion passengers and the age group to which most motorcyclists belong, the purpose of this study was to identify the spatial distribution of PYLL in the microregions of the state of Sao Paulo, according to accidents involving motorcycles from 2007 to 2011.

## METHODS

An exploratory ecological study was conducted using spatial analysis tools, on mortality data relating to accidents involving motorcycles in the state of São Paulo, Brazil, according to its 63 microregions. The mortality data were obtained from the Mortality Information System (SIM), which includes data from death certificates from all over the country and is available through the website of the Department of Information Technology of the Brazilian National Health System (DATASUS).^12^ The period examined was from January 1, 2007, to December 31, 2011, and the age groups covered were 20 to 29 years and 30 to 39 years. The diagnoses considered related to accidents involving motorcyclists (V20-V29 of the International Classification of Diseases, 10^th^ revision).

The sizes of the populations within these age strata (20-39 years of age) over this period were ascertained and mortality rates per 100,000 inhabitants were calculated, to make it possible to compare PYLL values between the microregions. Five-year periods were used to minimize possible fluctuations in death data. The population estimates used were obtained from the DATASUS website.^12^

The technique for PYLL estimation proposed by Romeder and McWhinnie was used,^13^ as expressed in the following mathematical formula:

PYLL = ∑(70 - i - 0.5) di

where *i* was taken to be the midpoint of the intervals from 20 to 29 years and from 30 to 39 years for each group, and *di* was the number of deaths in each microregion, according to age group.

The PYLL rates for the age groups of 20 to 29 years and 30 to 39 years were calculated to ensure comparability with other studies on populations of different sizes and populational pyramids. The following mathematical formula, adapted from Romeder and McWhinnie, was used:^13^

PYLL rate = (PYLL /N)*100,000 inhabitants

where N = the number of people in the corresponding age group.

The age limit of 70 years was chosen because this was the age proposed in the original method. 

These values were inserted in a digital map of the state of São Paulo according to its microregions, which was obtained from the Brazilian Institute for Geography and Statistics (Instituto Brasileiro de Geografia e Estatística, IBGE). Thematic maps were then constructed using the PYLL values for the ranges of 20-29 years and 30-39 years. In addition to the thematic maps, Kernel maps containing quartic function, density calculation, adaptive radius and 200 columns were constructed; these maps analyzed the behavior of dot patterns. Thus, through interpolation, the Kernel maps provided point intensities relating to the process, throughout the study region. From this, an overview of the areas of higher density of PYLL was obtained.^14^

Box maps were another form of graphical representation that was used. In this, correlations were made between Z (normalized values) and Wz values (averages of neighbors) in scatter plots divided into quadrants (Q). The Q values ranged from 1 to 4, such that Q1 corresponded to high-high values (i.e. high values of Z and high values of Wz); Q2, low-low; Q3, high-low; and Q4, low-high.^14^

The statistical analysis was based on calculation of the Global Moran Index (IM) for motorcycle riders’ PYLL rates. This index indicates the degree of spatial association in the information set, from the product to the mean, thus providing a single value as a measure of the spatial association for the entire data set. The values for this index, which is an overall measurement of spatial autocorrelation, are contained within the interval [-1; 1]. In calculating this index, it is important to establish its statistical validity by estimating its significance (P-value). Values close to zero indicate a lack of significant spatial autocorrelation between the values of the variables. Positive and negative values indicate positive or negative self-correlations between variables, respectively.^14^

The TerraView v4.2.2 public-access software developed by the National Institute of Space Research (Instituto Nacional de Pesquisas Espaciais, INPE) was used for the statistical analysis. Because this was an ecological study without the possibility that its subjects might be identified, it was not submitted to a research ethics committee for approval.

## RESULTS

A total of 5,348 deaths in the age range of 20 to 39 years over the years 2007 to 2011 were identified in the 63 microregions of the state of São Paulo. Of these, 3,686 deaths (68.9%) occurred in the age group of 20-29 years and 1,662 deaths (31.1%) in the age group of 30-39 years. There were 4,885 deaths of males (91.3%).

The mortality coefficients according to age group were 10.04 deaths/100,000 inhabitants for the range of 20-29 years and 4.96/100,000 inhabitants for the range of 30-39 years of age.

Table 1 shows that the mean PYLL rate among the population living in the state of São Paulo aged 20-29 years old was almost double that of the population aged 30-39 years. The minimum and maximum were also practically double in the 20-29 years population but the Moran indices for these age groups were not significantly different.

**Table 1:** Descriptive analysis on age group variables (years) with mean, standard deviation (SD), minimum (Min) and maximum (Max) values for potential years of life lost, and the Moran index (IM) with respective P-values, state of São Paulo, 2007-2011

Age group	Mean (SD)	Min-Max	IM (P-value)
20-29	486.9 (231.6)	98.1-1134.0	0.01 (0.41)
30-39	199.5 (105.5)	23.7-568.6	-0.04 (0.28)

**Figure 1:**
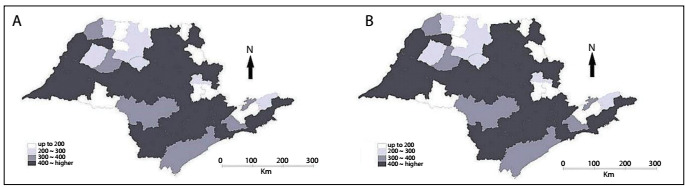
Rates of potential years of life lost per 100,000 inhabitants among motorcyclists aged 20-29 years (A) and 30-39 years (B), according to microregions of the state of São Paulo, 2007-2011.

Figure 1 shows the thematic maps of PYLL rates per 100,000 inhabitants for accidents involving motorcyclists aged 20-29 years (Figure 1A) and 30-39 years (Figure 1B). The highest contribution to these rates was the age group of 20 to 29 years, in which 38 microregions presented rates greater than 400 PYLL/100,000 inhabitants.

**Figure 2:**
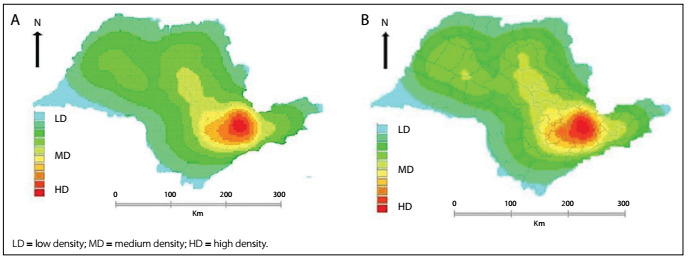
Kernel maps showing densities of rates of potential years of life lost per 100,000 inhabitants, among motorcyclists aged 20-29 years (A) and 30-39 years (B), according to microregions of the state of São Paulo, 2007-2011.

The Kernel maps show high densities of PYLL rates in microregions located in the metropolitan region of São Paulo and also in its surroundings, such as in Sorocaba, Osasco, Campinas, Jundiaí, Itapecerica da Serra, Franco da Rocha, Bragança Paulista and Piedade, for the age groups of 20-29 and 30-39 years (Figures 2A and 2B).

**Figure 3:**
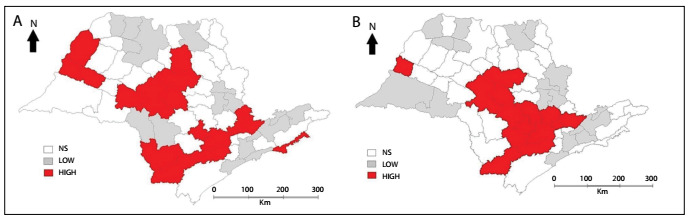
Box maps showing microregions that present high priority for interventions (HIGH), low priority (LOW) and no statistical significance (NS), according to potential years of life lost per 100,000 inhabitants, involving motorcyclists aged 20-29 years (A) and 30-39 years (B); state of São Paulo, 2007-2011.

Figure 3 presents Box maps that depict the microregions of high priority (HIGH), low priority (LOW) and no statistical significance (NS) regarding their PYLL rates per 100,000 inhabitants. Figure 3A shows the PYLL rates for the age group of 20 to 29 years old, and indicates that 17 microregions have high priority for interventions; while Figure 3B shows the PYLL rates for the age group of 30-39 years, and indicates that 14 microregions have high priority for interventions.

## DISCUSSION

To the best of our knowledge, this was the first study conducted using data from the state of São Paulo, thus making it difficult to compare this study with different places and times. In this study, greater concentration of accidents was identified in the metropolitan region of São Paulo and also in the microregions of Sorocaba, Osasco, Campinas, Jundiaí, Itapecerica da Serra, Franco da Rocha, Bragança Paulista and Piedade. Moreover, it was possible to identify microregions with high PYLL rates that deserve high-priority interventions to minimize these rates, to be implemented by regional managers and authorities linked to traffic management, given that these rates show the high impact of deaths within an economically active age range.

This study focused on a spatial approach to potential years of life lost by specifically addressing motorcycle accidents. Traffic accidents involving motorcycles in the state of São Paulo are worrying. In 2012, there were 1,700 deaths due to motorcycle accidents in the state of São Paulo and 1,200 (approximately 71%) occurred in the age group of 20-39 years, which is a young and economically active segment of the population. In our study, around 71% of the accidents involving motorcycles occurred among individuals in this age group, with higher prevalence among males (91.3%).^12^

The approach used here, i.e. spatial analysis, has been applied in many public health studies such as those describing accidents involving motorcyclists in Maringá, Paraná,^4^ and in the Paraíba valley, state of São Paulo,^6^ and in studies on other topics such as the spatial distribution of cesarean section rates in the state of São Paulo,^9^ Alzheimer’s deaths in the state of São Paulo^10^ and pneumonia in the Paraíba valley, state of São Paulo.^11^

A study conducted in the state of Pernambuco, in 2007, brought together spatial analysis and PYLL in relation to motorcycle accidents. It found that the mean number of PYLL due to RTA was 35.1 years, and that among the victims, 27.5% were motorcyclists, 24.6% were pedestrians and 17.82% were car occupants.^7^

In Rio Branco, Acre, the number of accidents involving motorcycles and the number of victims have been correlated with the growth of the fleet of motorcycles, which increased by 42.2% over the study period, while the fleet of other vehicle types increased by 9.2%. These accidents predominantly involved males and the age groups of 20 to 29 and 30 to 39 years.^15^

The Brazilian fleet of motorcycles increased from approximately 2,800,000 in 1998 to 16,500,000 in 2010, which was an increase of 490%.^2^ This increase, which resulted from marketing actions promoted by manufacturers and resellers, from low purchase prices associated with the introduction of long-term financing for instalment plans with small monthly repayments, and from use of motorcycles as a work tool for an increasing range of professional activities, may have contributed towards the increase in the number of accidents. Nevertheless, other causes may be correlated with this increase, including a lack of safety measures such as roads that are not always well maintained, riders’ carelessness in traffic, negligence regarding the quality and validity of helmets (which have a maximum useful life) and lack of supervision by the highway authorities regarding motorcycle and rider conditions.^1^^,^^4^

The data presented in this study coincide with the findings of Silva et al., who used spatial analysis in a study carried out in the state of Pernambuco, where the highest death rates involving motorcyclists formed urban clusters, including in the metropolitan region of Recife. Both studies showed higher rates of PYLL among in male motorcyclists and in the age group of 20-39 years.^16^

Our findings also coincide with those of Andrade and Mello-Jorge,^17^ who identified a loss of more than 1.3 million years due to terrestrial transport accidents over the years 2011 to 2013. They indicated that the age group that was most compromised was from 20 to 39 years, which accounted for 760,000 PYLL (around 60% of the total), of which 650,000 PYLL (around 86% in 20-39 age strata) related to males. The death rate per 100,000 inhabitants was highest (6.3) among motorcyclists. These authors showed that the costs were of the order of R$ 115 million (approximately US$ 30 million), which corresponded to 50% of the total expenses incurred through hospitalizations due to road traffic accidents and to 550,000 days of hospitalization due to traffic accidents involving motorcycles in 2012.^18^

Mascarenhas et al. evaluated records from emergency services in 24 state capitals and from the national capital (Brasília) relating to transportation accidents involving motorcyclists. They found that 66% of these admissions related to victims between 20 and 39 years of age, which was a rate similar to what was found in our study.^19^

In a time series study conducted using data from the state of Santa Catarina, among 19,889 motorcycle accidents, 84.43% of these involved male victims. The age groups of 20 to 29 years and 30 to 39 years accounted for a total of 10,564 hospitalizations (around 53%).^20^

Accidents involving motorcycles accounted for around 12% of all deaths in the age group 20-29 years old in our study. In total, males were much more affected (around 90%).^12^

The present study may have limitations and among these, it needs to be pointed out that the data used were secondary, although they were obtained from an official source (DATASUS). On the other hand, no information was available regarding whether deaths occurred at the accident site or at hospital shortly after the accident, or whether they occurred later on, as a result of complications from the injuries suffered by the motorcyclist. No information regarding whether the accidents occurred on highways or on the streets of the municipalities that make up the microregions studied was available. The systems compromised by the accident, such as skull trauma and fractures of upper and/or lower limbs, and the complications resulting from these injuries, have not been studied. In addition, ecological study designs do not indicate causality, but on the other hand they provide information to generate hypotheses. Nevertheless, the fact that microregions with high rates of lost years of life were identified through the present study is enough to activate regional and municipal managers to seek to discover the possible reasons that are leading to these disastrous findings.

## CONCLUSIONS

From the data of the present study, it can be concluded that the number of years of life lost through occurrences of motorcycle accidents in this young age group (20-39 years of age) constitutes an important social and public health problem. The spatial approach proved to be a useful tool for identifying microregions that deserve attention and provides backing for implementation of local and regional policies to minimize these accident rates.
